# Vaccination of Zoo Birds against West Nile Virus—A Field Study

**DOI:** 10.3390/vaccines11030652

**Published:** 2023-03-14

**Authors:** Felicitas Bergmann, Dominik Fischer, Luisa Fischer, Heike Maisch, Tina Risch, Saskia Dreyer, Balal Sadeghi, Dietmar Geelhaar, Lisa Grund, Sabine Merz, Martin H. Groschup, Ute Ziegler

**Affiliations:** 1 Friedrich-Loeffler-Institut, Federal Research Institute for Animal Health, Institute of Novel and Emerging Infectious Diseases, 17493 Greifswald-Insel Riems, Germany; 2 Der Gruene Zoo Wuppertal, Hubertusallee 30, 42117 Wuppertal, Germany; 3 Wildlife Research Institute, State Agency for Nature, Environment and Consumer Protection North Rhine-Westphalia, Puetzchens Chaussee 228, 53229 Bonn, Germany; 4 Thueringer Zoopark Erfurt, Am Zoopark 1, 99087 Erfurt, Germany

**Keywords:** West Nile virus (WNV), inactivated vaccine, zoological garden, zoo bird, Germany

## Abstract

West Nile virus (WNV) is known to cause disease and death in humans and various animals worldwide. WNV has circulated in Germany since 2018. In 2020, four birds tested positive for the WNV genome at Zoopark Erfurt (Thuringia). Moreover, virus neutralization assays detected neutralizing antibodies (nAb) against WNV in 28 birds. In addition, nAb against WNV and Usutu virus (USUV) were found in 14 birds. To protect valuable animals and to reduce the risk of viral transmission from birds to humans, we performed a field study on WNV vaccination at the zoo. To conduct the study, 61 birds from the zoo were categorized into three groups and subjected to a vaccination regimen, where each bird received either 1.0 mL, 0.5 mL, or 0.3 mL of a commercial inactivated WNV vaccine three times. The vaccinations were administered at three-week intervals, or as per modified vaccination schedules. Furthermore, 52 birds served as non-vaccinated controls. Adverse vaccination reactions were absent. The greatest increase in nAb titres was observed in birds that received 1.0 mL of vaccine. However, pre-existing antibodies to WNV and USUV appeared to have a major effect on antibody development in all groups and in all bird species, whereas sex and age had no effect. After vaccination, no death was detected in vaccinated birds for more than 1 year.

## 1. Introduction

West Nile virus (WNV) and Usutu virus (USUV) are members of the Flaviviridae family (genus *Flavivirus*) and belong to the Japanese encephalitis serogroup complex [1,2,3,4]. WNV may cause high morbidity and mortality in mammals and various bird species worldwide [5,6,7,8,9,10,11,12]. In birds, clinical signs associated with a WNV infection may range from mild, unspecific signs such as ruffled feathers, apathy, shedding of greenish excreta, and inappetence to severe neurological signs and fatalities. However, subclinical infections have also been reported [5,13,14]. In contrast to birds (amplifying hosts), humans and mammals including equids are considered to be dead-end hosts for WNV due to their low-level viremia [15,16]. WNV and USUV are transmitted by mosquitoes, and cycle in an enzootic arthropod-bird cycle in overlapping areas throughout Europe [7,17]. Both viruses share the same avian host range and a variety of identical arthropod vector species [3,17,18]. In Europe, USUV has been endemic for many years. In 2010, USUV was detected in Germany, first in mosquitoes, followed through mass die-offs in birds [19,20,21,22]. WNV was absent in continuous surveillance analysis in Germany until 2018, when it was detected in eastern Germany for the first time [23,24,25,26,27]. In the two following years, WNV continued to circulate and spread in bird and horse populations in the east of Germany [9,23,28]. 

WNV poses a health risk for humans and has been transmitted also via blood transfusions and organ transplants [29]. In 2021, four human WNV infections were confirmed in Germany as well as 34 in birds and 19 in horses [30,31]. To minimize the risk of viral transmission to humans and to interrupt transmission in the enzootic cycle in endemic regions, it is important to reduce the number of arthropod vectors [32], as well as the viral load in amplifying hosts. The latter may be achieved by vaccination of susceptible bird species. For this purpose, WNV vaccines are licensed only for use in horses in the European Union [33], thus effective treatments or commercial vaccines for birds and humans are not available. EQUIP WNV^®^ (Zoetis Belgium SA, Louvain-la-Neuve, Belgium) is a vaccine with inactivated West Nile virus lineage 1. Like the recombinant canarypox vaccine Proteq West Nile^®^ (Boehringer Ingelheim Vetmedica GmbH, Ingelheim am Rhein, Germany), this vaccine was proven to minimally reduce viremia and prevent severe disease in horses.

In the absence of licensed vaccines for birds, some bird holders and veterinarians in zoos have attempted to vaccinate endangered and threatened bird species against WNV using the equine vaccines, but the safety and efficacy of vaccination protocols are unknown [34]. Despite conducting some WNV vaccine trials in different avian species (e.g., penguins [34,35], flamingos [35,36,37,38], birds of prey [6,39], corvids [39], hawks [36,40], Galliformes [35], Falconiformes [41], and Passeriformes [42,43]), the results were not transferable to all species and have not been implemented in a prophylaxis concept of a zoological institution with a mixed avian collection. WNV vaccination protocols using different vaccines in different zoological facilities involve either two or three vaccinations for basic immunization at 14–21 days intervals, followed by annual boosters [34,44]. Davis et al., 2008 [34] conducted a study comparing a killed vaccine to a DNA vaccine and showed more robust and faster seroconversion in penguins vaccinated with the killed vaccine. Angenvoort et al., 2014 [41] compared two commercially available equine vaccines in falcons and showed a better protection when using a triple injection scheme compared to a double injection scheme for both vaccines. However, the side effects observed after using the recombinant vaccines led to the recommendation to use the inactivated vaccine. In another study, birds were vaccinated with an inactivated equine vaccine and showed best results after triple vaccination with 3-weekly intervals using 1.0 mL of the equine vaccine formulation [39]. However, serological results were lower when the birds were vaccinated with smaller volumes [36,39]. Furthermore, Olsen et al., 2009 [45] showed a weaker viremia and reduced cloacal virus shedding and lack of histological findings in the brain when the birds were vaccinated with a killed vaccine despite lack of seroconversion compared to unvaccinated birds. These studies suggest that vaccination is preferable in all cases and that the least side effects with the best possible protection can be expected from the use of inactivated equine vaccine. Okeson et al., 2007 [35] also discussed that different species may show different responses to vaccines; this could therefore be a reason for the different levels of seroconversion in different bird species. 

Zoopark Erfurt keeps various bird species such as the highly endangered Bali myna (*Leucopsar rothschildi*), northern bald ibis (*Geronticus eremita*), Eurasian griffon vulture (*Gyps fulvus*), snowy owl (*Bubo scandiacus*), and kea (*Nestor notabilis*) and has bred these animals as part of a European ex situ conservation program (EEP) run by EAZA (European Association of Zoos and Aquaria). The biosecurity concept includes testing and quarantine of all zoo birds. Furthermore, all dead zoo birds as well as all wild birds found dead in the zoo grounds are examined routinely for WNV, USUV, and other infectious pathogens at the local state laboratory office. During these investigations, the first case of WNV was confirmed in 2020 at the zoo. In total, four deceased birds were tested positive for WNV. In particular, WNV genomes were detected in (i) a female snowy owl by quantitative reverse transcription polymerase chain reactions (RT-qPCR) after it died on July 12th following several days of illness. (ii) One month later, signs of WNV infection were noticed in one of two conspecifics located in the same aviary and (iii) in a juvenile greater flamingo (*Phoenicopterus roseus*) prior to death. Furthermore, (iv) WNV was detected in an asymptomatically deceased greater flamingo. In addition to the WNV genome detection in zoo birds, WNV lineage 2 was found in eight mosquitoes and USUV (European lineages) in three mosquitoes trapped in the vicinity of the zoo in September [46]. At the same time, two male keas and one rainbow lorikeet (*Trichoglossus moluccanus*) showed clinical signs including apathy, beak lock, feather loss, reduced feed intake, and vomiting. These birds together with the remaining snowy owl were also tested for *Flaviviruses*, but neither the USUV genome nor the WNV genome could be detected in the blood. However, neutralizing antibodies (nAb) against WNV were found by WNV-specific virus neutralization test (VNT) in all animals. In the snowy owl, high antibody titres against both USUV and WNV were detected. The birds survived after treatment. In parallel, a natural and biological mosquito control in relevant biotopes was carried out in 2020 by reduction of water retention and using BTI (*Bacillus thuringiensis israelensis*) in open water areas, which was scientifically accomplished by the German Mosquito Control Association (KABS) [46].

The aim of the study was to reduce the zoonotic WNV risk for zoo visitors and staff by minimizing the infections and also the viral loads (viremia and virus excretion) in the birds potentially serving as amplifying hosts. At the same time, clinical diseases and animal losses in highly susceptible zoo birds should be prevented. In selected avian species, an inactivated WNV vaccine was used off-label in order to determine the innocuity, safety, and efficacy of this vaccine under field conditions. We also aimed to determine whether the inactivated WNV vaccine described above would reduce viremia and virus shedding in selected bird species kept at the zoo and whether the adjustment of the vaccination dose to the body weight would have an influence on the vaccination result. For this purpose, blood was collected from different avian species and analysed by molecular and serological assays. In addition, regular health checks of the zoo birds, including measuring of body weight, and close inspections of the vaccination sites were conducted.

## 2. Materials and Methods

### 2.1. Sample Collection

The trial was conducted from October 2020 to February 2022 and was flanked by mosquito control programs in 2020 and 2021 [46]. 

In October 2020, and at each vaccination timepoint (before the primary vaccination (0 days post vaccination (dpv)), 3 weeks after the primary vaccination (21 dpv), and 3 weeks after booster vaccination (42 dpv)), approximately 1.0 mL of whole blood was drawn and collected in serum separator medium (S-Monoject, Sarstedt) by metatarsal, jugular or ulnar venepuncture. In addition, blood samples were collected 3.5 weeks after third vaccination (69 dpv) and approximately 6 months later in early 2022 (261 dpv). Blood was separated into serum and blood coagulum by centrifugation and maintained at −20 °C until shipment on ice to the laboratories of the Friedrich-Loeffler-Institut.

### 2.2. Vaccination

The vaccine used in this study was EQUIP WNV^®^ (Zoetis Belgium SA, Louvain-la-Neuve, Belgium), a licensed emulsion for injection in horses. The commercially available vaccine is based on an inactivated WNV strain of lineage 1 (VM-2). It was stored at 4 °C until use. Birds with a body weight of >1000 g were administered 1.0 mL (recommended dose for horses), with a weight between 300 g and 1000 g 0.5 mL, or with a body weight <300 g 0.3 mL of the WNV vaccine intramuscular (i.m.) in the pectoral muscles on 0 dpv, 21 dpv, and 42 dpv (Table 1). Vaccination protocols were established for each bird species and individual depending on the body weight and the presence of existing WNV and USUV nAb titres in October 2020. Small-sized zoo birds belonging to Group A were vaccinated three times with 0.3 mL of the vaccine. Group B birds were vaccinated three times with 0.5 mL of the vaccine each time. Group C was divided into three subgroups. Birds in Group C.1. were vaccinated three times, in Group C.2. were vaccinated twice, and in Group C.3. were vaccinated only once with 1.0 mL vaccine. The control group (Group D) was not vaccinated but examined and sampled in the same way as Groups A, B, and C.1. Group D was divided into two subgroups, including 32 previously seronegative animals in subgroup D.1, and 20 previously seropositive animals in subgroup D.2 (Table 1). 

**Figure 1:**
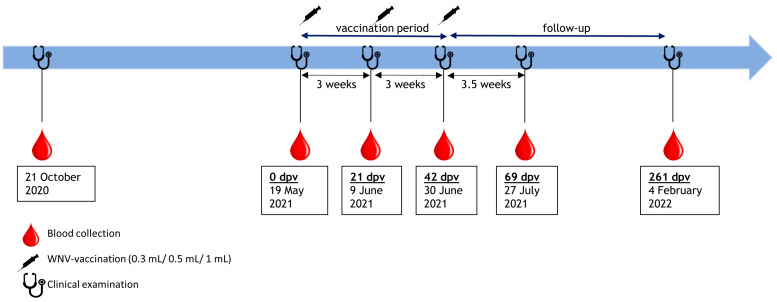
Timeline of the study period.

Clinical examination took place on each day of sampling. Furthermore, vaccination sites were thoroughly inspected and palpated on 21 dpv, 42 dpv, and 69 dpv for any evidence of unwanted reactions, such as abscesses or haematoma (Figure 1). 

### 2.3. Animals

In total, 113 susceptible zoo birds were included in the study belonging to 19 different bird species (Table 1). In total, 61 birds were vaccinated and 52 served as the non-vaccinated control with previously seronegative (Group D.1) and seropositive birds (Group D.2) (Table 1). Group D.1 included 31 birds that served as non-vaccinated controls and were sampled regularly; 11 birds that served as environmental controls and were sampled only in 2020; and ten offspring that were sampled only in July/February 2021/22 (Tables S5–S7). The selection of birds which received vaccination or served as controls was based on several criteria. As birds such as parrots, birds of prey/hawks, owls, ratites, and flamingos were classified highly susceptible to WNV infection, leading to disease and death, these species were vaccinated. Among others, the antibody status of all 85 sampled birds in autumn 2020 served as a benchmark. Bali mynas were not vaccinated, as there was no approval from the EEP species coordinator. In addition, less susceptible bird species such as ducks and chickens from the farm area were not vaccinated so that they could be used as sentinels for WNV circulation. Moreso, there were 20 greater flamingos among the controls that already had very high nAb titres against WNV and/or USUV at baseline in autumn 2020. 

**Figure 2:**
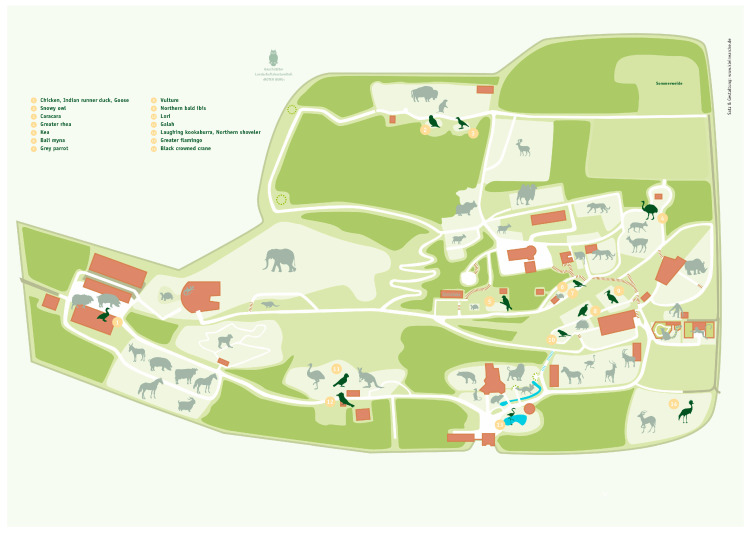
Bird aviary locations at Zoopark Erfurt, Germany.

Animals were kept at Zoopark Erfurt, Germany in outdoor aviaries distributed throughout the zoo (Figure 2). All birds were monitored daily by zookeepers and examined regularly by the zoo veterinarian. Prior to vaccination and sampling, it was ensured that birds were clinically healthy. In order to not disturb the birds’ breeding behaviour, vaccination and sampling was carried out outside of the species-specific breeding period. Consequently, the timing of the second and third vaccination and the follow up blood collections varied among the avian species, individuals, and groups and deviated from the standardised schedules of the field experiment (Table 1). A three-dose regimen (1.0 mL/dose, 3-weeks apart) was followed except for those birds belonging to Group A and B, which received only 0.3 mL or 0.5 mL, respectively. Vaccination procedures and timepoints of each animal are depicted in Table 1.

### 2.4. Diagnostic Methods

Molecular biological and serological methods were applied to test all zoo birds for both WNV and USUV RNA by RT-qPCRs [22,47]. Viral RNA of coagulum was extracted using RNeasy^®^ Mini Kit (Qiagen, Hilden, Germany) following the manufacturer’s instructions. WNV-RT-qPCR was performed according to a previously published protocol using primers and probes which target 118 base pairs in the 5′-untranslated region (UTR) [47]. USUV-RT-qPCR was performed (as described by [22]) which targets the non-structural protein 1 gene. Together with all samples, an internal control RNA (IC RNA) containing 2 × 10^5^ copies/µL was extracted and included as a duplex RT-qPCR [48].

Commercially available blocking WNV IgG enzyme-linked immunosorbent assays (bELISA) (INgezim^®^ West Nile Compac, Ingenasa, Madrid, Spain) were performed on serum samples from all zoo birds included in this study to allow species-independent detection of WNV antibodies against the Pr-E envelope protein, following the manufacturer’s instructions. Samples were considered positive when the inhibition percentage (IP) was >40%, doubtful with IP ≥ 30% to ≤40%, and negative with IP < 30%. 

To validate bELISA results, WNV-VNTs were performed under biosafety level 3 conditions using Vero cells on 96-well plates as described previously [24,26]. WNV strain Germany (lineage 2, GenBank accession No. MH924836) was used to quantify cross-reacting antibodies among the Japanese encephalitis serogroup. As USUV was also circulating in Zoopark Erfurt in 2020 and may lead to serological cross reactions, it was always tested in parallel. USUV strain Germany (Europa 3, GenBank accession No. HE599647) was used following the same protocol. Sera from experimentally infected animals or hyperimmune sera from vaccinated animals with known WNV and USUV antibody titres, as well as serum which was antibody negative for both viruses, were included as positive and negative controls, respectively. Neutralising antibody titres were calculated according to the Behrens–Kaerber method [49]. The maximum dilution was defined as the neutralization dose 50% (ND_50_) of a sample at which the cytopathic effect was inhibited in 50% of wells [49]. Serum samples were considered positive with ND_50_ values equal to or above 10, and negative with ND_50_ values below 10. Sera were considered specific if only one of the viruses was neutralized or ND_50_ titres were ≥4 times higher for one virus [50]. In the presence of similarly high antibody titres to both viruses, the result often has to be interpreted as inconclusive, as it is difficult to distinguish between WNV- and USUV-specific nAb due to cross-reactivity. The antibody titres may be interpreted with greater certainty as the outcome of co-infection with both viruses if the following situation exists: the neutralizing titres to both viruses are on a very high level and there is a detectable high infection pressure from both viruses in an overpopulated area where many susceptible species live simultaneously. This is the fact for the area of the Zoopark Erfurt. A similar situation of possible co-infection with WNV and USUV was recently described for the area of zoological facilities in Berlin and Halle [18].

### 2.5. Sequencing and Phylogenetic Analysis

All sampled zoo birds were screened for the presence of WNV and USUV by RT-qPCR. In case of a positive WNV-RT-qPCR result, whole genome sequencing was performed using MinION by Oxford Nanopore-technology (Oxford Science Park, the United Kingdom) similar to that previously described for USUV [51]. In short, complementary DNA (cDNA) was synthesised by multiplex PCR using SuperScript IV First-Strand cDNA Synthesis Reaction (Cat. no. 18091050; Invitrogen by Thermo Fisher Scientific, Darmstadt, Germany) using random primers (Invitrogen by Thermo Fisher Scientific, Darmstadt, Germany)) for reverse transcription (described by [52]). Followed by an amplification step performed with primers previously published by [53]. The PCR product was purified with Agencourt AMPure XP beads (Agencourt, Beckmann-Coulter, United States). The barcoding and ligation mixes were prepared using the NEBNext Ultra II End Repair/dA-Tailing Module, NEBNext Ultra II Ligation Module, and NEBNext Quick Ligation Module (New England Biolabs, Ipswich, MA, USA), 1D Native Barcoding Genomic DNA Kit (with EXP-NBD104 and SQK-LSK109; ONT), and Flow Cell Priming Kit (EXP-FLP002; ONT), following the manufacturer’s instructions. The sequencing was performed on a MinION MK1c instrument, using an R9.4.1 spot-on flow cell.

Basecalling, demultiplexing, and adaptor trimming were performed using Guppy in the MK1C sequencer. For consensus sequence generation, mapping with reference genomes was conducted using Minimap2 [54]. Consensus sequence was analysed using BLASTn with default settings [55].

### 2.6. Statistical Analysis

For this study, the following variables were collected: species, treatment groups including vaccination volume and vaccination interval, age in years, gender, and finally the neutralizing antibody titres. To test for potential interactions between the variables, analysis of variance was conducted using the ANOVA and Generalized Linear Model (GLM) procedures. For each species, Fisher’s exact test was used to make comparisons of titre status between experimental groups. ANCOVA (analysis of the covariance) models was used for statistical comparison of the slopes of two regression lines. Statistical analysis was performed using SPSS software (IBM Corp. Released 2011, IBM SPSS Statistics for Windows, Version 20.0, IBM Corporation, Armonk, NY, USA). *p*-value < 0.05 was considered statistically significant.

### 2.7. Ethical Statement

The animals were kept at the zoological institution according to European husbandry guidelines and national animal welfare regulations. The experiment was permitted by the Thuringian State Office for Consumer Protection (reference number: 2684-04-15-ZOO-20-101; approved 14 September 2020).

## 3. Results

### 3.1. Adverse Reactions of Vaccination

No adverse reactions were observed in any of the vaccinated birds. No abnormalities of skin or muscle were noticed at the injection site nor was there any weight loss. The behaviour of the vaccinated birds was inconspicuous and the feed intake remained unchanged. Despite the potential stress associated with capturing and holding the birds, as well as bleeding and vaccinating them, the breeding populations of greater flamingos, laughing kookaburras, and rainbow lorikeets in 2021 successfully raised offspring. Chicks also hatched during and after the study in the non-vaccinated Group D.1 (geese and crested chickens). Only one greater rhea died in connection with capture prior to vaccination and blood collection. A full necropsy in the deceased bird revealed excitation-induced cardiovascular arrest of the animal as the likely cause of death, which is a common condition in ratites. 

### 3.2. Molecular Biological Results

No WNV or USUV genomes were detected in 437 of 438 blood coagula from overall 113 birds (different collection time points) (see Figure 1). However, one rainbow lorikeet (No. 19) tested positive for WNV-RNA by WNV-specific RT-qPCR (Cycle threshold (Ct) value 27.68) on 19 May 2021. Whole genome sequencing (Accession number: OQ326499) was performed and revealed WNV lineage 2 of Eastern German clade (new classification as subcluster 2.5.3.4.3c), which also circulated in the zoo in 2020 [56]. The bird showed no clinical signs. Except for this finding, virus circulation in the zoo was not confirmed in 2021 [57].

### 3.3. Serological Results

In 2020, 28 birds revealed specific nAb against WNV. A total of 5 zoo birds showed specific nAb against USUV and 38 birds had not seroconverted at all. In addition, 14 birds had high specific nAb titres against both WNV and USUV, suggesting a double infection with both viruses in the past (Table S1). The results of serology regarding the formation of specific antibodies after vaccination varied greatly, mostly depending on the applied vaccination regimen and on the presence of nAb from a previous infection. Therefore, the results were evaluated separately according to the amount of vaccine application and the frequency of vaccinations. However, in all vaccinated birds, age and sex had no effect on antibody development (*p*-value > 0.05).

#### 3.3.1. Group A—0.3 mL Dose Regimen Vaccinated Three Times

**Figure 3:**
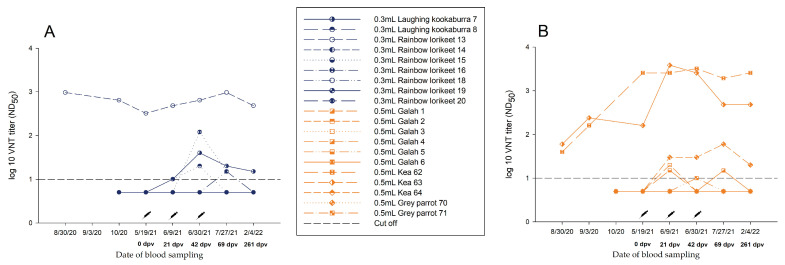
Neutralizing antibodies in Group A and Group B, inoculated three times with (**A**) 0.3 mL (blue) or (**B**) 0.5 mL (yellow), detected by virus neutralization test (VNT) during vaccination period.

Antibody development throughout the study period was not statistically significant *(p*-value > 0.05). After the second vaccination, a slight increase in nAb titre specific for WNV was noted in four of nine birds that had no WNV-Ab prior to vaccination. However, at 69 dpv, and even more pronounced at 261 dpv, a drop-in antibody was detected in all birds (Figure 3A, Table S2). 

One rainbow lorikeet (No. 13) already had high nAb titres in 2020 (August: ND_50_ 1/960; October: ND_50_ 1/640). During winter, until the start of the vaccination study in May 2021, the nAb titre decreased (ND_50_ 1/320). As a result of the triple vaccination, it reached a titre of ND_50_ 1/960 in July 2021 (Figure 3A, Table S2).

#### 3.3.2. Group B—0.5 mL Dose Regimen Vaccinated Three Times

In three of nine birds without pre-existing antibodies, a slight nAb titre increase specific for WNV was measured after the second vaccination. At the time of the follow-up at 261 dpv, a similar drop in titre was evident in the birds, comparable to Group A (Figure 3B, Table S3). No statistical differences in titre progression were determined (*p*-value > 0.05). 

By comparison, two keas (Nos. 62, 63) that already had high WNV nAb titres (No. 62: ND_50_ 1/160; No. 63: ND_50_ 1/240) at the start of the vaccination study in 2020, retained at a similar level through the triple vaccination. However, the increase in antibodies in 2021 in response to vaccination was not significant (*p*-value > 0.05) (Figure 3B, Table S3).

#### 3.3.3. Group C.1—1.0 mL Dose Regimen Vaccinated Three Times

**Figure 4:**
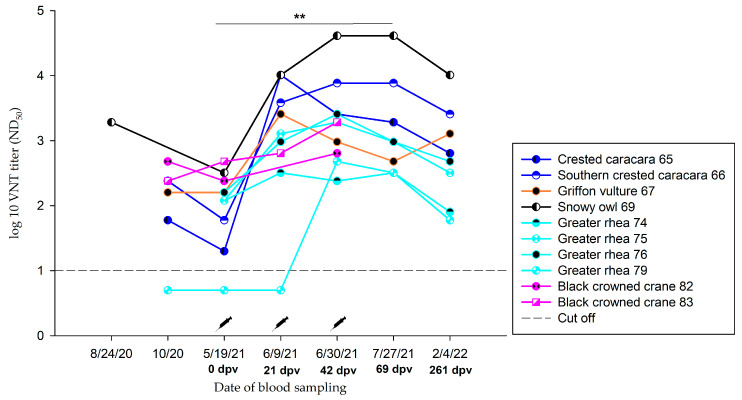
Neutralizing antibodies in Group C.1, inoculated three times with 1.0 mL vaccine, detected by virus neutralization test (VNT) during vaccination period. **: Significant, *p*-value < 0.05.

Birds vaccinated three times with 1.0 mL demonstrated a significant increase in nAb specific for WNV after vaccination (*p*-value < 0.05). However, the majority of the vaccinated birds already had nAb against WNV at the beginning of the vaccination study in May 2021 (Table S4). These titres remained at a high level (ND_50_ ≥ 1/60) throughout the study period (Figure 4, Table S4). Two greater flamingos that were antibody negative prior to vaccination (0 dpv) are described in detail in Section 3.3.7. 

#### 3.3.4. Group C.2—1.0 mL Dose Regimen Vaccinated Twice

**Figure 5:**
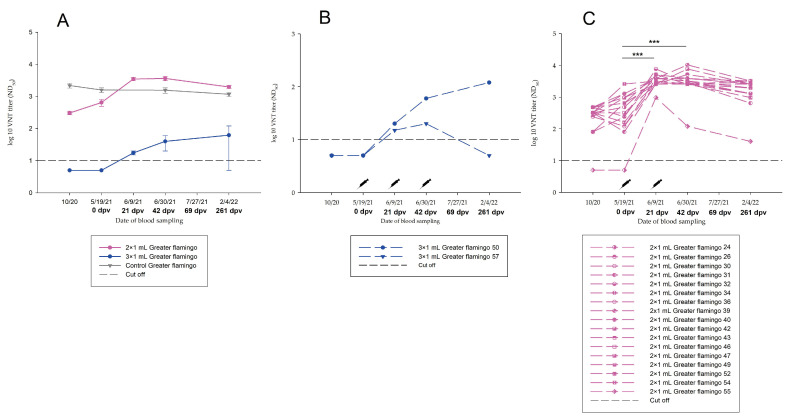
Neutralizing antibodies in all greater flamingos (Group C.1, C.2, D.2) tested during the study period, detected by virus neutralization test (VNT) during vaccination period. (**A**) The average value of all sampled flamingos. (**B**) The individual values of all flamingos vaccinated three times (Group C.1). (**C**) The individual values of all flamingos vaccinated twice (Group C.2). ***: Extremely significant, *p*-value < 0.0001. Rosa: greater flamingos vaccinated twice with 1.0 mL (Group C.2); blue: greater flamingos vaccinated three times with 1.0 mL (Group C.1); grey: non-vaccinated control group of greater flamingos (Group D.2).

In all greater flamingos vaccinated twice with 1.0 mL, a significant increase in nAb specific for WNV was detected after vaccination (*p*-value < 0.05). Importantly, the majority of vaccinated birds had nAb specific for WNV at the start of vaccination (Table S4). Again, these titres remained at a high level throughout the study period, although a significant decrease in antibody response was observed between 69 dpv and 261 dpv (*p*-value < 0.05). One greater flamingo (No. 55) tested negative for WNV-specific nAb at the start of vaccination also seroconverted successfully after two vaccinations (ND_50_ 1/960), although at a comparably low titre level (Table S4, Figure 5C).

#### 3.3.5. Group C.3—1.0 mL Dose Regimen Vaccinated Once

Northern bald ibis were vaccinated only once with 1.0 mL. Due to the presence of already high specific nAb for WNV before vaccination (Table S4), a booster effect nevertheless set in after vaccination. WNV nAb titres remained at high levels throughout the study period, although no statistically significant differences were observed (*p*-value > 0.05) (Figure S1, Table S4). 

#### 3.3.6. Group D—Control Group without Vaccination

All 20 greater flamingos (Group D.2) with pre-existing WNV nAb that served as non-vaccinated controls showed almost consistently high WNV nAb titres through the study period, with no statistically significant variation in titres (*p*-value > 0.05). The remaining 32 non-vaccinated animals (Group D.1) without pre-existing WNV antibodies showed no detectable WNV nAb throughout the study period (Tables S5–S7). 

#### 3.3.7. Summary of the Serological Results of Greater Flamingos

In total, 39 greater flamingos were sampled during the study period. Twenty greater flamingos with pre-existing high antibody titres served as the non-vaccinated control (Group D.2). In this manner, it was possible to follow the progression of nAb in naturally infected greater flamingos over 1 year (Figure 5A). The remaining 19 greater flamingos were vaccinated (Group C.1, C.2). Two of them belonged to Group C.1 and received three vaccinations, whereas 17 belong to Group C.2 and received only two vaccinations due to pre-existing WNV antibodies before vaccination (Figure 5). Antibody titres of Group C.2, which were vaccinated twice, increased significantly over the study period (*p*-value < 0.05) (Figure 5C). In contrast, antibodies in two greater flamingos that were antibody negative prior to vaccination (0 dpv) and were vaccinated three times (Group C.1) did not increase significantly (*p*-value > 0.05). One flamingo had nAb detectable throughout the study period, while the other greater flamingo (No. 57) remained the exception, with no nAb detectable at 261 dpv (Figure 5B). The variation in antibody development in these groups might be a result of a baseline difference in antibodies against WNV and USUV (Figure 5). All flamingos belonging to Group C.1 had no WNV antibodies detectable prior to vaccination. Antibody development during the vaccination period was not significant, and absolute antibody titres did not reach the high level of Group C.2. In comparison, most flamingos in Group C.2 had already existing antibodies to WNV prior to vaccination, ranging from ND_50_ 1/80 to ND_50_ 1/480. In addition, high USUV titres were detected in two birds. Moreover, five greater flamingos had high titres against both, USUV and WNV. An exception in Group C.2 is the great flamingo No. 55, in which no antibodies were present prior to vaccination. Due to the strong increase in antibodies after the second vaccination (69 dpv), this individual was vaccinated only twice. It is evident that the drop-in antibody titre was greater at 261 dpv, compared to the other birds belonging to Group C.2 (Figure 5C). In most cases, antibody titres of vaccinated flamingos were higher than titres of unvaccinated flamingos (Figure 5A). In conclusion, vaccination in greater flamingos leads to a significant increase in titre (*p*-value < 0.05).

### 3.4. Usutu Virus Circulation in the Zoo

**Figure 6:**
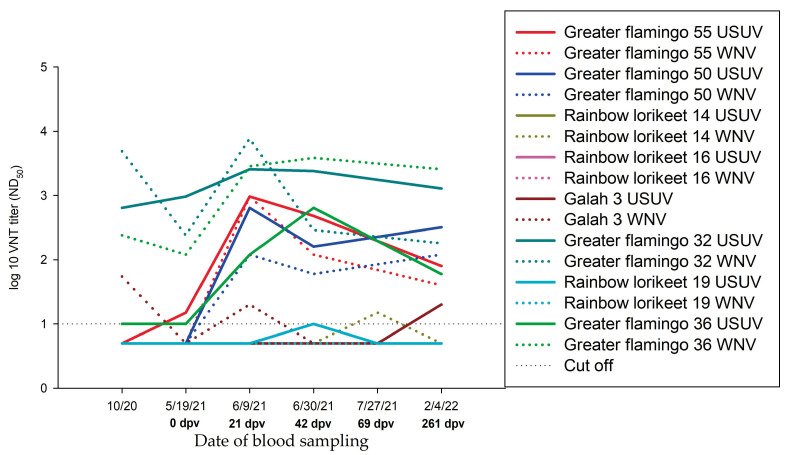
Neutralizing antibodies in zoo birds with an assumed USUV infection, detected by virus neutralization test (VNT) during study period. Bold: nAb to USUV; dashed: nAb to WNV.

In 2020, USUV infections occurred in the Zoopark Erfurt [46]. There is evidence that USUV circulated in 2021 as well. Unfortunately, there is no molecular biological confirmation for USUV infection available but results of serological examinations allow this estimation. Figure 6 shows samples of the antibody courses of some zoo birds in which an USUV infection is assumed because of an observed seroconversion in animals. For completeness, the titres of both USUV and WNV antibodies are depicted, because cross-reactions between the two *Flaviviruses* are known to occur [58], as well as co-infections [18].

## 4. Discussion

Birds in zoological gardens, especially those participating in international species conservation programs, have a high conservation and economic value. WNV infections in birds can lead to severe and fatal disease, and birds of several avian species, including birds of prey, ratites, and parrots, which are often kept in zoos, are highly susceptible [13,14,42,59,60,61,62,63,64,65]. To date, there is no WNV vaccine licensed for use in any avian species and scant data are available on antibody responses in bird species following the use of commercially available vaccines. The aim of the present study was therefore to evaluate the safety of a WNV vaccine in zoo birds and its efficacy in protecting against the deadly disease [42,59]. 

In present study, all birds were vaccinated with an inactivated equine WNV vaccine (commercially available from Zoetis). Birds that received 1.0 mL showed better antibody responses (Group C) (Figure 4 and Figure 5, Table S4) compared to those that were vaccinated using a smaller amount of vaccine. Birds vaccinated with 0.3 mL (Group A) showed no significant increase in WNV-antibody titres (Figure 3A, Table S2). The same was observed for titres of birds without pre-existing antibodies that received an amount of 0.5 mL (Group B) (Figure 3B, Table S3). 

Antibody titres of individual birds differed, sometimes considerably after vaccination with a killed vaccine. This is consistent with previous studies that showed no seroconversion in flamingos vaccinated with 0.2 mL but did with 1.0 mL [35]. Olsen et al., 2009 [45] did not demonstrate elevated antibody titres in sandhill cranes (*Grus canadensis*); however, vaccination appears to provide some protection that leads to a faster increase in titres after challenge. Beside the direct reaction to the vaccine, a wide variety of factors could explain the different immune responses following vaccination. As the virus is known to have circulated in the zoo the year before vaccination [46,56], natural exposure to the virus may explain a rise in antibody titres, which limits interpretation of titres. Accordingly, it cannot be ruled out that the virus was also circulating in the zoo in early 2021, although there is no evidence from virus isolation from birds or mosquitos in 2021 (personal communication by N. Becker). However, these factors must be regarded as unpreventable influences in field studies where settings and environment differ significantly from certified animal trial laboratories. It is likely that a continuous biological mosquito control implemented since 2020 [46] has reduced the number of mosquitoes in the zoo, thus reducing the risk of spreading mosquito-borne diseases. In the context of the birds’ positive response to vaccination, a reduction in the risk of virus transmission to staff and visitors can be assumed. On 19 May, prior to vaccination, the WNV genome was detected in a rainbow lorikeet (No. 19) and a seroconversion was detected in a greater flamingo (No. 49). However, during the course of the study, all negative control birds (Group D.1) remained seronegative. Likewise, offspring of the zoo birds remained negative (Table S6). Even so, juveniles are considered particularly susceptible to infections due to their immature and naive immune system [38].

As a result of the co-circulation of USUV and WNV in the Zoopark Erfurt in 2020 [46], high antibody titres against both viruses were found in many birds (Table S1). It is known that co-circulation occurs in terms of geographic range, host, and vector species [3,9,18] and even co-infections can be found [18]. The high nAb titres observed against both WNV and USUV may be a rare finding due to sequential or simultaneous infections caused by the high infection pressure for both flaviviruses. Additionally, there is a close antigenic relationship between WNV and USUV [66]. The high antibodies caused by infection with closely related flaviviruses such as WNV and USUV may result in cross-reactivity and challenge test interpretation. [24,27]. Both envelope proteins of the two viruses are very similar which suggests a potential cross-reactivity and cross-immunity [67,68]. Antibody-dependent enhancement in dengue virus infections has been reported in the literature, but to date there is no evidence that this phenomenon also occurs in WNV [69,70,71,72,73]. It is more likely that these cross-reacting antibodies can confer cross-protection with another and perhaps even against infections with other related flaviviruses [60,70,74,75]. Therefore, there is a possibility that the WNV-associated mortality rate in the zoological garden in 2020 was alleviated due to previous USUV infections. In October 2020, the greater flamingos displayed significantly elevated levels of antibodies to both viruses. In several instances, the antibodies levels were so high that it was challenging to distinguish between the specificities of USUV and WNV, as indicated in Table S1. This hypothesis of cross-protection is also supported by the results of a previous study [70] showing that mice pre-infected with USUV are protected from disease and death when subsequently infected with WNV.

One reason for the large differences in antibody development, apart from pre-existing antibodies, is the extent of vaccination. The avian species in the present study were divided into three different vaccination groups receiving different vaccine doses based on their size and body weights: Group A received 0.3 mL, Group B 0.5 mL, and Group C 1.0 mL vaccine emulsion. This contrasts to previous studies, where 1.0 mL of an inactivated vaccine was applied intramuscularly (e.g., penguins [34,35], flamingos [35,36], Attwater’s prairie chickens (*Tympanuchus cupido attwateri*) [35], large falcons (*Falco* spp.) [41], birds of prey and corvids [39], and hawks [36]). However, in these studies only larger sized birds were vaccinated and no birds below 500 g or even below 300 g, as in the present study. In Group A and B with 0.3 mL and 0.5 mL, respectively, no significant increase in antibody titres (*p*-value > 0.05) was demonstrated, whereas vaccination with 1.0 mL (Group C) resulted in a significant increase (*p*-value < 0.05). A similar observation was made in flamingos when 0.2 mL of the vaccine did not elicit a measurable immune response [36], but 1.0 mL did [35]. However, a comparably low vaccine dose of 0.5 mL stimulated an efficient protection against WNV-challenge in similarly sized sandhill cranes (*Grus canadensis*) [45]. 

Species-specific differences may have resulted in different antibody progressions as well. In previous studies, it has been shown that vaccination may result in varying levels of seroconversion depending on the vaccinated avian species. Davis et al., 2008 [34] showed that serological results post-vaccination vary widely across different penguin species, which was confirmed in penguins and flamingos [35]. This reinforces the assumption that vaccination volumes can have different effects in different species. Therefore, the outcome of the present study is influenced also by these two variables (species, vaccine dose) and their individual impacts cannot be judged essentially. One option to increase antibody titres in birds of Group A and B might have been to generally increase the vaccination volumes. However, when considering animal welfare aspects, such large volumes cannot be administered into the pectoralis muscle of small sized birds. Administering such a comparably large dose into both sides of the pectoral muscles or injecting part of the volume subcutaneously instead of intramuscularly would perhaps be a solution. Furthermore, it would have been interesting to find out if an annual booster shot would have increased the titre progression [44]. Therefore, species-specific studies and adaptations of the vaccination scheme are necessary in the future.

In horses, vaccination is recommended 1 to 2 months before the start of the arthropod season [33]. Likewise, vaccination may be recommended in birds in WNV endemic areas. However, the minimal levels of antibody titres that were able to effectively protect against clinical disease and death are unknown for most avian species. Therefore, no guideline on the best possible vaccination timing can be given. The gold standard for evaluating vaccine efficacy would be a post-vaccination challenge using WNV, but this is not possible for endangered and highly valuable zoo birds [34]. In addition to the nAb, other factors of the avian immune response are known to play an important role in the defence against infections [76]. Therefore, looking only at serological results depicts only the humoral immune system, but not the innate and cellular responses. However, in birds, knowledge about the involvement of cellular immunity in protection against WNV is limited. In contrast, studies in mammals show that cell-mediated immunity is important for the protection against WNV associated diseases [77,78,79]. Therefore, similar mechanisms may be expected in birds as well.

In total, 39 greater flamingos were sampled during the study period belonging to Group C.1, C.2 and D.1, depending on their pre-existing antibodies. Antibody titres of Group C.2, with WNV nAb ranging from ND_50_ 1/80 to ND_50_ 1/480, increased significantly over the study period (*p*-value < 0.05). In contrast, the progression of antibodies in greater flamingos without WNV nAb prior to vaccination (Group C.1) did not increase significantly (*p*-value > 0.05) (Figure 5, Table S4). Therefore, the presence of pre-existing antibodies (to both WNV and USUV) may be a reason for a stronger response to vaccination (Figure 5).

A plausible reason for the consistently high titres of non-vaccinated flamingos (Group D.2) during the study period may be a possible consequence of very high nAb titres against WNV and USUV, as well as co-protection based on past co-infections with both viruses. High nAb titres to both flaviviruses were described in zoological facilities, where co-infections were detected on a molecular level [18]. In naturally infected pigeons, the presence of WNV antibodies was shown over a period of > 15 months [80]. This study demonstrated the presence of antibodies in naturally infected flamingos, ranging between ND_50_ 1/160 and ND_50_ 1/2560 over a period of more than 16 months. Furthermore, it cannot be excluded completely that re-infections with both WNV and USUV occurred during the study period although no genome was found during the vaccination period.

Age and sex had no effect on antibody progression (*p*-value > 0.05) which is consistent with results of other studies [34]. After vaccination, no clinical signs were observed at any time of the study. No unusual behaviours, health changes, or adverse reactions were observed in any of the birds. Therefore, the inactivated vaccine appeared to be safe in all avian species used in this study. These results are compatible to previous studies [34,35,36,41], in which no adverse reactions or unusual behaviour were observed with the use of an inactivated vaccine.

## 5. Conclusions

The inactivated vaccine was safe in all avian species treated in this study, as no adverse reactions were observed. In addition, vaccination with 1.0 mL of inactivated vaccine at 3-week intervals resulted in good antibody responses in most zoo bird species. However, pre-existing antibodies against WNV and USUV seem to strongly enhance antibody formation through vaccination. It can be assumed that the zoonotic WNV risk for zoo visitors and staff was reduced.

## Figures and Tables

**Table 1:** Zoo birds belonging to the different study groups. Vaccination volume and frequency for each vaccinated avian species. Non-vaccinated controls including offspring and environmental controls.

Group	Species Order	Common Name	Scientific Name	No. Birds	1st Vaccination	2nd Vaccination	3rd Vaccination
A	*Coraciiformes*	Laughing kookaburra	*Dacelo novaeguineae*	2	0.3 mL	0.3 mL	0.3 mL
A	*Coraciiformes*	Rainbow lorikeet	*Trichoglossus haematodus*	7	0.3 mL	0.3 mL	0.3 mL
B	*Psittaciformes*	Galah	*Eolophus roseicapilla*	6	0.5 mL	0.5 mL	0.5 mL
B	*Psittaciformes*	Kea	*Nestor notabilis*	3	0.5 mL	0.5 mL	0.5 mL
B	*Psittaciformes*	Grey parrot	*Psittacus erithacus*	2	0.5 mL	0.5 mL	0.5 mL
C.1	*Falconiformes*	Southern crested caracara	*Caracara plancus*	1	1.0 mL	1.0 mL	1.0 mL
C.1	*Falconiformes*	Crested caracara	*Caracara cheriway*	1	1.0 mL	1.0 mL	1.0 mL
C.1	*Accipitriformes*	Griffon vulture	*Gyps fulvus*	1 *	1.0 mL	1.0 mL	1.0 mL
C.1	*Rheiformes*	Greater rhea	*Rhea americana*	4	1.0 mL	1.0 mL	1.0 mL
C.1	*Gruiformes*	Black crowned crane	*Balearica pavonina*	2	1.0 mL	1.0 mL	1.0 mL
C.1	*Strigiformes*	Snowy owl	*Bubo scandiacus*	1	1.0 mL	1.0 mL	1.0 mL
C.1	*Phoenicopteriformes*	Greater flamingo	*Phoenicopterus roseus*	2	1.0 mL	1.0 mL	1.0 mL
C.2	*Phoenicopteriformes*	Greater flamingo	*Phoenicopterus roseus*	17	1.0 mL	1.0 mL	NV
C.3	*Pelecaniformes*	Northern bald ibis	*Geronticus eremita*	13	1.0 mL	NV	NV
D.1	*Rheiformes*	Greater rhea	*Rhea americana*	1	NV	NV	NV
D.1	*Anseriformes*	Northern shoveler	*Spatula clypeata*	5	NV	NV	NV
D.1	*Galliformes*	Thuringer chicken	*Gallus gallus domesticus*	5	NV	NV	NV
D.1	*Galliformes*	Sebright bantam chicken	*Gallus gallus domesticus*	3	NV	NV	NV
D.1	*Anseriformes*	Indian runner duck	*Anas platyrhynchos f. domestica*	4	NV	NV	NV
D.1	*Anseriformes*	Steinbach fighting goose	*Anser anser f. domestica*	4	NV	NV	NV
D.1	*Passeriformes*	Bali myna	*Leucopsar rothschildi*	2	NV	NV	NV
D.1	*Galliformes*	Common peafowl	*Pavo cristatus*	2	NV	NV	NV
D.1	*Coraciiformes*	Rainbow lorikeet	*Trichoglossus haematodus*	1	NV	NV	NV
D.1	*Coraciiformes*	Laughing kookaburra	*Dacelo novaeguineae*	4	NV	NV	NV
D.1	*Accipitriformes*	Griffon vulture	*Gyps fulvus*	1	NV	NV	NV
D.2	*Phoenicopteriformes*	Greater flamingo	*Phoenicopterus roseus*	20	NV	NV	NV

Abbreviation: NV = not vaccinated; * 4th vaccination on 6 October 2021.

## Data Availability

The data that supports the findings of this study are available in the main manuscript and the Supplementary Materials of this article.

## Supplementary Materials

The following supporting information can be downloaded at: https://www.mdpi.com/article/10.3390/vaccines11030652/s1, Figure S1: Neutralizing antibodies in Northern bald ibis (Group C.3) vaccinated once with 1.0 mL, detected by virus neutralization test (VNT) during vaccination period.; Table S1: Results of WNV and USUV neutralization assays from birds sampled in 2020; Table S2: WNV and USUV neutralization assay results in all tested birds vaccinated with 0.3 mL vaccine (Group A); Table S3: WNV and USUV neutralization assay results in all tested birds vaccinated with 0.5 mL vaccine (Group B); Table S4: WNV and USUV neutralization assay results in all tested birds vaccinated with 1.0 mL vaccine (Group C); Table S5: WNV and USUV neutralization assay results in all non-vaccinated control birds (Group D); Table S6: WNV and USUV neutralization assay results in offspring (Group D); Table S7: WNV and USUV neutralization assay results in environmental controls (Group D).

## References

[1] Engler O., Savini G., Papa A., Figuerola J., Groschup M.H., Kampen H., Medlock J., Vaux A., Wilson A.J., Werner D. (2013). European surveillance for West Nile virus in mosquito populations. Int. J. Environ. Res. Public Health.

[2] Campbell G.L., Marfin A.A., Lanciotti R.S., Gubler D.J. (2002). West Nile virus. Lancet Infect. Dis..

[3] Nikolay B. (2015). A review of West Nile and Usutu virus co-circulation in Europe: How much do transmission cycles overlap?. Trans. R. Soc. Trop. Med. Hyg..

[4] De Madrid A.T., Porterfield J.S. (1974). The flaviviruses (group B arboviruses): A cross-neutralization study. J. Gen. Virol..

[5] Chancey C., Grinev A., Volkova E., Rios M. (2015). The global ecology and epidemiology of West Nile virus. Biomed Res. Int..

[6] Chang G.-J.J., Davis B.S., Stringfield C., Lutz C. (2007). Prospective immunization of the endangered California condors (*Gymnogyps californianus*) protects this species from lethal West Nile virus infection. Vaccine.

[7] Kramer L.D., Styer L.M., Ebel G.D. (2008). A global perspective on the epidemiology of West Nile virus. Annu. Rev. Entomol..

[8] Pietsch C., Michalski D., Münch J., Petros S., Bergs S., Trawinski H., Lübbert C., Liebert U.G. (2020). Autochthonous West Nile virus infection outbreak in humans, Leipzig, Germany, August to September 2020. Eurosurveill.

[9] Ziegler U., Bergmann F., Fischer D., Müller K., Holicki C.M., Sadeghi B., Sieg M., Keller M., Schwehn R., Reuschel M. (2022). Spread of West Nile Virus and Usutu Virus in the German Bird Population, 2019–2020. Microorganisms.

[10] Yeung M.W., Shing E., Nelder M., Sander B. (2017). Epidemiologic and clinical parameters of West Nile virus infections in humans: A scoping review. BMC Infect. Dis..

[11] Wünschmann A., Shivers J., Bender J., Carroll L., Fuller S., Saggese M., van Wettere A., Redig P. (2005). Pathologic and immunohistochemical findings in goshawks (*Accipiter gentilis*) and great horned owls (*Bubo virginianus*) naturally infected with West Nile virus. Avian Dis..

[12] Lanciotti R.S., Roehrig J.T., Deubel V., Smith J., Parker M., Steele K., Crise B., Volpe K.E., Crabtree M.B., Scherret J.H. (1999). Origin of the West Nile virus responsible for an outbreak of encephalitis in the northeastern United States. Science.

[13] Komar N., Langevin S., Hinten S., Nemeth N., Edwards E., Hettler D., Davis B., Bowen R., Bunning M. (2003). Experimental infection of North American birds with the New York 1999 strain of West Nile virus. Emerg. Infect. Dis..

[14] Ziegler U., Angenvoort J., Fischer D., Fast C., Eiden M., Rodriguez A.V., Revilla-Fernández S., Nowotny N., de La Fuente J.G., Lierz M. (2013). Pathogenesis of West Nile virus lineage 1 and 2 in experimentally infected large falcons. Vet. Microbiol..

[15] Troupin A., Colpitts T.M. (2016). Overview of West Nile Virus Transmission and Epidemiology. Methods Mol. Biol..

[16] McLean R.G., Ubico S.R., Bourne D., Komar N. (2002). West Nile virus in livestock and wildlife. Curr. Top. Microbiol. Immunol..

[17] Zannoli S., Sambri V. (2019). West Nile Virus and Usutu Virus Co-Circulation in Europe: Epidemiology and Implications. Microorganisms.

[18] Santos P.D., Michel F., Wylezich C., Höper D., Keller M., Holicki C.M., Szentiks C.A., Eiden M., Muluneh A., Neubauer-Juric A. (2021). Co-infections: Simultaneous detections of West Nile virus and Usutu virus in birds from Germany. Transbound. Emerg. Dis..

[19] Weissenböck H., Kolodziejek J., Url A., Lussy H., Rebel-Bauder B., Nowotny N. (2002). Emergence of Usutu virus, an African mosquito-borne flavivirus of the *Japanese encephalitis* virus group, central Europe. Emerg. Infect. Dis..

[20] Becker N., Jöst H., Ziegler U., Eiden M., Höper D., Emmerich P., Fichet-Calvet E., Ehichioya D.U., Czajka C., Gabriel M. (2012). Epizootic emergence of Usutu virus in wild and captive birds in Germany. PLoS ONE.

[21] Cadar D., Lühken R., van der Jeugd H., Garigliany M., Ziegler U., Keller M., Lahoreau J., Lachmann L., Becker N., Kik M. (2017). Widespread activity of multiple lineages of Usutu virus, western Europe, 2016. Eurosurveill.

[22] Jöst H., Bialonski A., Maus D., Sambri V., Eiden M., Groschup M.H., Günther S., Becker N., Schmidt-Chanasit J. (2011). Isolation of usutu virus in Germany. Am. J. Trop. Med. Hyg..

[23] Ziegler U., Lühken R., Keller M., Cadar D., van der Grinten E., Michel F., Albrecht K., Eiden M., Rinder M., Lachmann L. (2019). West Nile virus epizootic in Germany, 2018. Antivir. Res..

[24] Michel F., Sieg M., Fischer D., Keller M., Eiden M., Reuschel M., Schmidt V., Schwehn R., Rinder M., Urbaniak S. (2019). Evidence for West Nile Virus and Usutu Virus Infections in Wild and Resident Birds in Germany, 2017 and 2018. Viruses.

[25] Ziegler U., Jöst H., Müller K., Fischer D., Rinder M., Tietze D.T., Danner K.-J., Becker N., Skuballa J., Hamann H.-P. (2015). Epidemic Spread of Usutu Virus in Southwest Germany in 2011 to 2013 and Monitoring of Wild Birds for Usutu and West Nile Viruses. Vector-Borne Zoonotic Dis..

[26] Seidowski D., Ziegler U., von Rönn J.A.C., Müller K., Hüppop K., Müller T., Freuling C., Mühle R.-U., Nowotny N., Ulrich R.G. (2010). West Nile virus monitoring of migratory and resident birds in Germany. Vector-Borne Zoonotic Dis..

[27] Michel F., Fischer D., Eiden M., Fast C., Reuschel M., Müller K., Rinder M., Urbaniak S., Brandes F., Schwehn R. (2018). West Nile Virus and Usutu Virus Monitoring of Wild Birds in Germany. Int. J. Environ. Res. Public Health.

[28] Bergmann F., Trachsel D.S., Stoeckle S.D., Bernis Sierra J., Lübke S., Groschup M.H., Gehlen H., Ziegler U. (2022). Seroepidemiological Survey of West Nile Virus Infections in Horses from Berlin/Brandenburg and North Rhine-Westphalia, Germany. Viruses.

[29] Petersen L.R. (2019). Epidemiology of West Nile Virus in the United States: Implications for Arbovirology and Public Health. J. Med. Entomol..

[30] Frank C., Schmidt-Chanasit J., Ziegler U., Lachmann R., Preußel K., Offergeld R. (2022). West Nile Virus in Germany: An Emerging Infection and Its Relevance for Transfusion Safety. Transfus. Med. Hemother..

[31] Friedrich-Loeffler-Institut West-Nil-Virus. https://www.fli.de/de/aktuelles/tierseuchengeschehen/west-nil-virus/.

[32] Nasci R.S., Mutebi J.-P. (2019). Reducing West Nile Virus Risk Through Vector Management. J. Med. Entomol..

[33] StIKoVet Stellungnahme zur Immunisierung von Pferden gegen das West-Nil-Virus. https://www.openagrar.de/servlets/MCRFileNodeServlet/openagrar_derivate_00017232/Stellungnahme_WNV-Impfung_Pferde_2018-10-22.pdf.

[34] Davis M.R., Langan J.N., Johnson Y.J., Ritchie B.W., van Bonn W. (2008). West Nile virus seroconversion in penguins after vaccination with a killed virus vaccine or a DNA vaccine. J. Zoo Wildl. Med..

[35] Okeson D.M., Llizo S.Y., Miller C.L., Glaser A.L. (2007). Antibody Response of Five Bird Species after Vaccination with a Killed West Nile Virus Vaccine. J. Zoo Wildl. Med..

[36] Nusbaum K.E., Wright J.C., Johnston W.B., Allison A.B., Hilton C.D., Staggs L.A., Stallknecht D.E., Shelnutt J.L. (2003). Absence of humoral response in flamingos and red-tailed hawks to experimental vaccination with a killed West Nile virus vaccine. Avian Dis..

[37] Siegal-Willott J.L., Carpenter J.W., Glaser A.L. (2006). Lack of Detectable Antibody Response in Greater Flamingos (*Phoenicopterus ruber ruber*) after Vaccination against West Nile Virus with a Killed Equine Vaccine. J. Avian Med. Surg..

[38] Baitchman E.J., Tlusty M.F., Murphy H.W. (2007). Passive Transfer of Maternal Antibodies to West Nile Virus in Flamingo Chicks (*Phoenicoperius Chilensis* and *Phoenicopterus Ruber Ruber*). J. Zoo Wildl. Med..

[39] Johnson S. (2005). Avian titer development against West nile virus after extralabel use of an equine vaccine. J. Zoo Wildl. Med..

[40] Redig P.T., Tully T.N., Ritchie B.W., Roy A.F., Baudena M.A., Chang G.-J.J. (2011). Effect of West Nile virus DNA-plasmid vaccination on response to live virus challenge in red-tailed hawks (*Buteo jamaicensis*). Am. J. Vet. Res..

[41] Angenvoort J., Fischer D., Fast C., Ziegler U., Eiden M., de La Fuente J.G., Lierz M., Groschup M.H. (2014). Limited efficacy of West Nile virus vaccines in large falcons (*Falco* spp.). Vet. Res..

[42] Bertelsen M.F., Olberg R.-A., Crawshaw G.J., Dibernardo A., Lindsay L.R., Drebot M., Barker I.K. (2004). West Nile virus infection in the eastern loggerhead shrike (*Lanius ludovicianus migrans*): Pathology, epidemiology, and immunization. J. Wildl. Dis..

[43] Wheeler S.S., Langevin S., Woods L., Carroll B.D., Vickers W., Morrison S.A., Chang G.-J.J., Reisen W.K., Boyce W.M. (2011). Efficacy of three vaccines in protecting Western Scrub-Jays (*Aphelocoma californica*) from experimental infection with West Nile virus: Implications for vaccination of Island Scrub-Jays (*Aphelocoma insularis*). Vector-Borne Zoonotic Dis..

[44] Glavis J., Larsen R.S., Lamberski N., Gaffney P., Gardner I. (2011). Evaluation of antibody response to vaccination against West Nile virus in thick billed parrots (*Rhynchopsitta pachyrhyncha*). J. Zoo Wildl. Med..

[45] Olsen G.H., Miller K.J., Docherty D.E., Bochsler V.S., Sileo L. (2009). Pathogenicity of West Nile virus and response to vaccination in sandhill cranes (*Grus canadensis*) using a killed vaccine. J. Zoo Wildl. Med..

[46] Fynmore N., Lühken R., Maisch H., Risch T., Merz S., Kliemke K., Ziegler U., Schmidt-Chanasit J., Becker N. (2021). Rapid assessment of West Nile virus circulation in a German zoo based on honey-baited FTA cards in combination with box gravid traps. Parasit. Vectors.

[47] Eiden M., Vina-Rodriguez A., Hoffmann B., Ziegler U., Groschup M.H. (2010). Two new real-time quantitative reverse transcription polymerase chain reaction assays with unique target sites for the specific and sensitive detection of lineages 1 and 2 West Nile virus strains. J. Vet. Diagn. Investig..

[48] Hoffmann B., Depner K., Schirrmeier H., Beer M. (2006). A universal heterologous internal control system for duplex real-time RT-PCR assays used in a detection system for pestiviruses. J. Virol. Methods.

[49] Mayr A., Bachmann P.A., Bibrack B., Wittmann G. (1977). Neutralisationstest: Virologische Arbeitsmethoden (Serologie).

[50] Yeh J.-Y., Lee J.-H., Park J.-Y., Seo H.-J., Moon J.-S., Cho I.-S., Kim H.-P., Yang Y.-J., Ahn K.-M., Kyung S.-G. (2012). A diagnostic algorithm to serologically differentiate West Nile virus from *Japanese encephalitis* virus infections and its validation in field surveillance of poultry and horses. Vector-Borne Zoonotic Dis..

[51] Holicki C.M., Bergmann F., Stoek F., Schulz A., Groschup M.H., Ziegler U., Sadeghi B. (2022). Expedited retrieval of high-quality Usutu virus genomes via Nanopore sequencing with and without target enrichment. Front. Microbiol..

[52] Quick J., Grubaugh N.D., Pullan S.T., Claro I.M., Smith A.D., Gangavarapu K., Oliveira G., Robles-Sikisaka R., Rogers T.F., Beutler N.A. (2017). Multiplex PCR method for MinION and Illumina sequencing of Zika and other virus genomes directly from clinical samples. Nat. Protoc..

[53] Sikkema R.S., Schrama M., van den Berg T., Morren J., Munger E., Krol L., van der Beek J.G., Blom R., Chestakova I., van der Linden A. (2020). Detection of West Nile virus in a common whitethroat (*Curruca communis*) and Culex mosquitoes in The Netherlands, 2020. Eurosurveill.

[54] Li H. (2018). Minimap2: Pairwise alignment for nucleotide sequences. Bioinformatics.

[55] National Library of Medicine, National Center for Biotechnology Information Basic Local Alignment Search Tool (BLAST). Version 2.13.0. https://blast.ncbi.nlm.nih.gov/Blast.cgi.

[56] Santos P.D., Günther A., Keller M., Homeier-Bachmann T., Groschup M.H., Beer M., Höper D., Ziegler U. (2022). An advanced sequence clustering and designation workflow reveals the enzootic maintenance of a dominant West Nile virus subclade in Germany. bioRxiv.

[57] Friedrich-Loeffler-Institut West-Nil-Virus Auch in der Saison 2021 Wieder Aktiv. https://www.fli.de/de/aktuelles/kurznachrichten/neues-einzelansicht/west-nil-virus-auch-in-der-saison-2021-wieder-aktiv/.

[58] Poidinger M., Hall R.A., Mackenzie J.S. (1996). Molecular characterization of the *Japanese encephalitis* serocomplex of the flavivirus genus. Virology.

[59] Ludwig G.V., Calle P.P., Mangiafico J.A., Raphael B.L., Danner D.K., Hile J.A., Clippinger T.L., Smith J.F., Cook R.A., McNamara T. (2002). An outbreak of West Nile virus in a New York City captive wildlife population. Am. J. Trop. Med. Hyg..

[60] Nemeth N.M., Bosco-Lauth A.M., Bowen R.A. (2009). Cross-protection between West Nile and *Japanese encephalitis* viruses in red-winged blackbirds (*Agelaius phoeniceus*). Avian Dis..

[61] Nemeth N.M., Kratz G.E., Bates R., Scherpelz J.A., Bowen R.A., Komar N. (2008). Naturally induced humoral immunity to West Nile virus infection in raptors. Ecohealth.

[62] Wodak E., Richter S., Bagó Z., Revilla-Fernández S., Weissenböck H., Nowotny N., Winter P. (2011). Detection and molecular analysis of West Nile virus infections in birds of prey in the eastern part of Austria in 2008 and 2009. Vet. Microbiol..

[63] Carboni D.A., Nevarez J.G., Tully T.N., Evans D.E. (2008). West Nile virus infection in a sun conure (*Aratinga solstitialis*). J. Avian Med. Surg..

[64] Burt F.J., Grobbelaar A.A., Leman P.A., Anthony F.S., Gibson G.V.F., Swanepoel R. (2002). Phylogenetic relationships of southern African West Nile virus isolates. Emerg. Infect. Dis..

[65] Fischer D., Muir A., Aparici Plaza D., Herrmann K., Pynnonen-Oudman K. (2019). EAZA Usutu and West Nile Virus Management Guidelines.

[66] Calisher C.H., Karabatsos N., Dalrymple J.M., Shope R.E., Porterfield J.S., Westaway E.G., Brandt W.E. (1989). Antigenic relationships between flaviviruses as determined by cross-neutralization tests with polyclonal antisera. J. Gen. Virol..

[67] Nikolay B., Fall G., Boye C.S.B., Sall A.A., Skern T. (2014). Validation of a structural comparison of the antigenic characteristics of Usutu virus and West Nile virus envelope proteins. Virus Res..

[68] Oliphant T., Nybakken G.E., Engle M., Xu Q., Nelson C.A., Sukupolvi-Petty S., Marri A., Lachmi B.-E., Olshevsky U., Fremont D.H. (2006). Antibody recognition and neutralization determinants on domains I and II of West Nile Virus envelope protein. J. Virol..

[69] Thomas S., Redfern J.B., Lidbury B.A., Mahalingam S. (2006). Antibody-dependent enhancement and vaccine development. Expert Rev. Vaccines.

[70] Blázquez A.-B., Escribano-Romero E., Martín-Acebes M.A., Petrovic T., Saiz J.-C. (2015). Limited susceptibility of mice to Usutu virus (USUV) infection and induction of flavivirus cross-protective immunity. Virology.

[71] Sinigaglia A., Pacenti M., Martello T., Pagni S., Franchin E., Barzon L. (2019). West Nile virus infection in individuals with pre-existing Usutu virus immunity, northern Italy, 2018. Eurosurveill.

[72] Porterfield J.S. (1986). Antibody-Dependent Enhancement of Viral Infectivity. Adv. Virus Res..

[73] Percivalle E., Cassaniti I., Sarasini A., Rovida F., Adzasehoun K.M.G., Colombini I., Isernia P., Cuppari I., Baldanti F. (2020). West Nile or Usutu Virus? A Three-Year Follow-Up of Humoral and Cellular Response in a Group of Asymptomatic Blood Donors. Viruses.

[74] Price W.H., Thind I.S. (1972). The mechanism of cross-protection afforded by dengue virus against West Nile virus in hamsters. J. Hyg..

[75] Tesh R.B., Arroyo J., Da Travassos Rosa A.P.A., Guzman H., Xiao S.-Y., Monath T.P. (2002). Efficacy of killed virus vaccine, live attenuated chimeric virus vaccine, and passive immunization for prevention of West Nile virus encephalitis in hamster model. Emerg. Infect. Dis..

[76] Gerlach H. (1994). Avian Medicine: Principles and Application: Defense Mechanisms of the Avian Host.

[77] Brien J.D., Uhrlaub J.L., Nikolich-Zugich J. (2007). Protective capacity and epitope specificity of CD8(+) T cells responding to lethal West Nile virus infection. Eur. J. Immunol..

[78] Engle M.J., Diamond M.S. (2003). Antibody prophylaxis and therapy against West Nile virus infection in wild-type and immunodeficient mice. J. Virol..

[79] Purtha W.E., Myers N., Mitaksov V., Sitati E., Connolly J., Fremont D.H., Hansen T.H., Diamond M.S. (2007). Antigen-specific cytotoxic *T lymphocytes* protect against lethal West Nile virus encephalitis. Eur. J. Immunol..

[80] Gibbs S.E.J., Hoffman D.M., Stark L.M., Marlenee N.L., Blitvich B.J., Beaty B.J., Stallknecht D.E. (2005). Persistence of antibodies to West Nile virus in naturally infected rock pigeons (*Columba livia*). Clin. Diagn. Lab. Immunol..

