# Supplementary figures and images for: Vaccination of Zoo Birds against West Nile Virus—A Field Study
Source: Vaccines (Basel). 2023 Mar 14;11(3):652. doi: 10.3390/vaccines11030652 (PMC10058624; doi:10.3390/vaccines11030652)

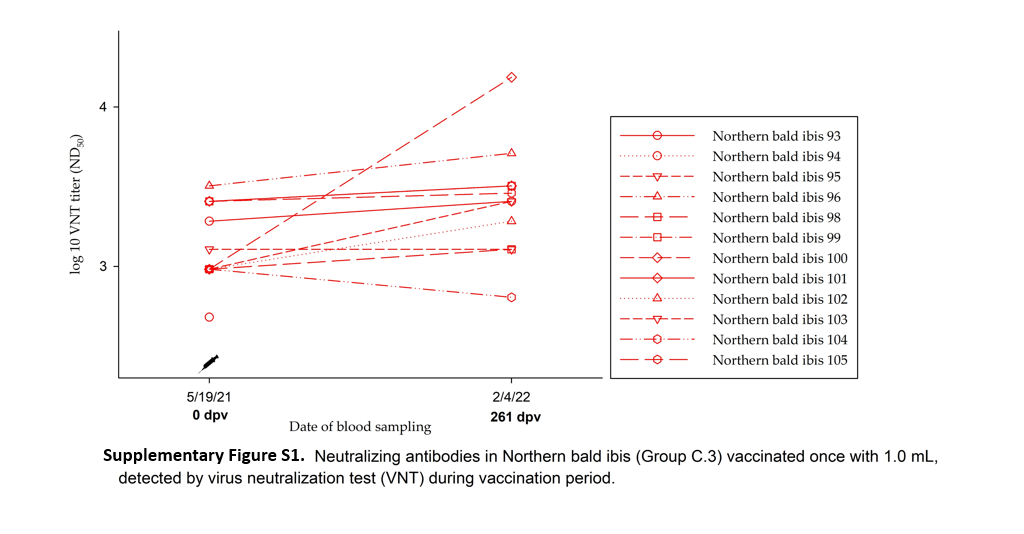

Supplement: Supplementary file 1 [file vaccines-11-00652-s001.zip › Supplemental Figure S1.png]
